# Exploring the Volatile Profile of *Vanilla planifolia* after Fermentation at Low Temperature with *Bacillus* Isolates

**DOI:** 10.3390/foods13172777

**Published:** 2024-08-30

**Authors:** Thabani-Sydney Manyatsi, Yu-Hsin Lin, Pin-Hui Sung, Ying-Tzy Jou

**Affiliations:** 1 Department of Tropical Agriculture and International Cooperation, National Pingtung University of Science and Technology, Neipu Shuefu Road 1, Pingtung 91201, Taiwan; thabanimanyatsi@gmail.com; 2 Department of Biological Science and Technology, National Pingtung University of Science and Technology, Neipu Shuefu Road 1, Pingtung 91201, Taiwan; bobo428769@gmail.com; 3 Kaohsiung District Agricultural Research and Extension Station, Ministry of Agriculture, Dehe Road 2-6, Pingtung 90846, Taiwan; phsung@mail.kdais.gov.tw

**Keywords:** vanilla, bacterial fermentation, GC-MS, volatile compounds, vanillin

## Abstract

*Vanilla planifolia* is grown as a high-value orchid spice for its odor and savor attributes that increase due to the curing process associated with microbial colonization. This tends to influence the aromatic properties of vanilla. Hence, 11 *Bacillus* sp. strains were isolated from *V. planifolia* and identified with 16S rRNA gene sequencing. The liquid culture (1 mL of 10^7^ CFU mL^−1^) of selected *Bacillus vallismortis* NR_104873.1:11-1518, *Bacillus velezensis* ZN-S10, and *Bacillus tropicus* KhEp-2 effectively fermented green-blanched vanilla pods kept at 10 °C during the sweating stage. GC-MS analysis showed that the methanol extract of non-coated, and *B. vallismortis* treated vanilla detected three (3) volatile compounds, whereas seven (7) components were obtained in *B. tropicus* and *B. velezensis* treatment. 4H-pyran-4-one, 2,3-dihydro-3,5-dihydroxy-6-methyl was found in *B. velezensis* ZN-S10, *B. tropicus* KhEp-2, and *B. vallismortis* while it was not present in the control samples. This ketone compound suggested a Maillard reaction resulting in brown-increased aroma pods. Linoleic acid and Hexadecanoic acid ethyl esters were detected only in ZN-S10 strain-coated vanilla. A novel 3-Deoxy-d-mannoic lactone was detected only in *B. vallismortis*-treated vanilla characterized as a new compound in *V. planifolia* which suggested that the new compound can be altered with the coating of bacteria in vanilla during fermentation. Thus, the *Bacillus* strains improved the volatile profile and exhibited a new aroma and flavor profile of vanilla owing to bacteria fermentation during the curing process.

## 1. Introduction

Vanilla (*Vanilla planifolia*) is denoted as one of the worldwide high-value orchids grown as a spice after saffron and as an herbaceous bean pod [1,2,3]. Vanilla extracts are essential in food as flavor and aroma agents, along with other applications in fragrances, pharmaceuticals, and aromatherapy [4,5,6]. The demand for vanilla has been escalating for the past decades. Still, recent reports have shown that the quantity of natural vanilla tends to be inadequate owing to the propagation, harvest, and post-harvest challenges farmers and processors face [5,7,8]. Green-harvested vanilla bean pods must undergo a curing process for aromatic production while yielding natural vanillin [4,9]. Blanching is the first step performed during the curing of vanilla, followed by sweating or fermentation. Proper moisture levels (≈60%) are maintained to prevent microbial spoilage while allowing enough moisture content for enzyme-catalyzed processes, which might produce molds in pods [9,10]. Hence, to halt mold formation, the sweating or fermenting vanilla pods are then dried further in the sun or by air, wherein later, the conditioning stage is carried out for 2–3 months to obtain highly-flavored, brown, and cured vanilla beans with the moisture of about 20–35% water [11]. Fermentation at low temperatures (10–15 °C) has been reported to be effective in eliminating spoilage microorganisms while allowing the production of volatile compounds in fermented foods [12,13]. According to studies, the conditioned pods produce improved flavors of sweet, flowery, smoked, spicy, sweet, and prune-to-raisin vanilla beans [4].

Vanillin (4-hydroxy-3-methoxybenzaldehyde) is the crucial critical compound in vanilla beans among more than 200 compounds found in *Vanilla planifolia* [14,15]. These components are responsible for the flavor and aroma characteristics, which include 4-methoxybenzyl alcohol, acetic acid, and 1,3-octadiene [16,17]. Studies have shown that microbes are crucial to the synthesis of vanillin. For instance, the colonization of the microbes in cured vanilla beans increased the vanillin content and other volatile compounds responsible for the aroma and flavor development [15,18]. The microbial distribution observed with different isolation techniques, such as morphological analysis and 16S rRNA gene sequencing methods, have been reported to be involved in bacterial and fungal communities, including *Bacillus*, *Enterobacter* sp., *Citrobacter* sp., and *Pseudomonas* [19,20]. Due to the propagation of vanilla beans in the field, microbes accumulate during the microbial growth until the harvest period. This expansion produces many metabolites at post-harvest treatment with a curing process [21]. Xu et al. [22] have also reported that communities of bacteria are involved in the curing correlated with fungal microorganisms. Specifically, the development of the vanilla flavor was developed primarily due to the role of *Bacillus* sp. as the critical responsible candidate of microbes. The flavor formation was also reported to be influenced by *Aspergillus*, which was observed to have the highest relative abundance of these microbes during the conditioning step of curing blanched *Vanilla planifolia* Andrews. Hence, the development of the vanilla odor and flavor can be influenced by the role of microbes tangled in the curing procedure.

The physicochemical and microbial properties are significantly influenced throughout the curing process of *V. planifolia,* resulting in altered aroma and flavor attributes [23]. Hence, other studies have demonstrated that a variety of methods can be used to analyze and quantify the components found in vanilla, such as high-pressure liquid chromatography (HPLC) [24], and gas chromatography-mass spectrometry (GC-MS) [25] methods. Headspace solid-phase microextraction (HS-SPME) consisting of a GC flame ionization detector (GC-FID) [15], reversed-phase liquid chromatography (RPLC) [26,27], and near-infrared spectroscopy (NIR) [28] techniques have also been used by other researchers. Similarly, according to Gu et al. [29], employing an HPLC–MS showed that vanillyl alcohol, capsaicin, glucose, and cresol are broadly dispersed in the microbial metabolism involved in vanillin production. It should be noted that most researchers have shown that the index quality of vanilla can be determined by the content and ratio of volatile components such as vanillin and guaiacol [30,31,32]. Studies have analyzed ˃ 60 volatile compounds with the GC–MS method wherein the vanilla bean pods were coated with *Bacillus subtilis* subsp. *subtilis* for an effective fermentation of the pods [33]. Researchers have also stipulated that using *Bacillus* isolates on sweating or fermenting vanilla beans can be a simple, cheap, and developing technique compared to impractical biotic elicitors or enzyme-assisted methods for large-scale natural vanillin production [34].

According to studies, vanilla extracts comprise volatile compounds with vanillin playing a critical role in the sensory attributes due to major aroma and flavor changes caused by this component [35]. Henceforth, an analysis of the volatile compounds has been of interest in this study to upsurge the understanding of the flavor and aroma characteristics in *V. planifolia* after treatment of the pods with edible *Bacillus* bacteria culture during the fermentation stage of the curing process. Furthermore, this study analyzed vanillin and other volatile compounds using GC-MS techniques on the cured vanilla pods. It should be noted that the isolated bacteria were obtained from naturally cured vanilla bean pods. *Bacillus vallismortis* NR_104873.1:11-1518, *Bacillus velezensis* ZN-S10, and *Bacillus tropicus* KhEp-2 were the designated bacterial strains coated in green blanched vanilla bean pods for the fermentation or sweating of the *V. planifolia* compared with non-bacterial treated samples (control).

## 2. Materials and Methods

### 2.1. Plant Material and Curing

**Figure 1:**
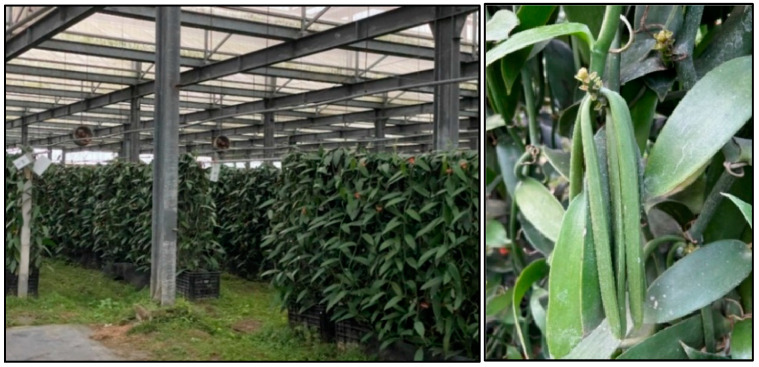
Vanilla plants in a greenhouse (**left**) in Pingtung, Taiwan, being harvested as green matured vanilla pods (**right**) used as samples for this study.

The isolation of bacteria from vanilla (*Vanilla planifolia*) and other experiments were performed at the National Pingtung University of Science and Technology (NPUST) with green-matured vanilla pods (Figure 1) harvested from Pingtung farm in the southern region of Taiwan (22°25′41.8″ N 120°32′29.0″ E). Washing and sorting according to the length and size of the vanilla bean were performed immediately after the samples reached the NPUST Department of Biological Science and Technology laboratory facilities. Blanching of the vanilla was conducted with hot water (80 °C) immersion for 2 min to sterilize and remove undesired microorganisms. The *V. planifolia* samples were subsequently dried with paper towels and allowed to sweat or ferment in plastic boxes for 2 days at room temperature. The pods were then placed in a humidified freezer of 10 °C and air dried for 2 h every 3 days for about 3 weeks during the sweating or fermentation stage. The conditioning stage dried the pods at a low temperature of 10 °C for color alterations from yellow brown to dark brown indicating cured vanilla. The process took about 3 months before the isolation of bacteria. Low-temperature fermentation (at 10 °C) and conditioning were aimed at edible microorganisms’ selection during bacteria purification.

### 2.2. Bacteria Culture Preparation and Isolation

In this study, we sampled the bacteria isolates from naturally fermented vanilla bean pods by culturing cut samples. Conventionally cured vanilla pods (0.5 cm dried cuts) were soaked as 0.5 g powder at 20 °C overnight in sterile distilled water in 15 mL Falcon tubes after sonication in an ultrasonic bath (Delta Model DC 150-H, Takahashi, Okayama, Japan) for 20 min. As per modifications from the technique by Chen et al. [20], the supernatant (500 µL) was pipetted onto MRS agar plates. The MRS medium comprises 15 g L^−1^ dextrose, 2.5 g L^−1^ agar, yeast extract (5 g L^−1^), 2 g L^−1^ of potassium phosphate, magnesium sulfate (0.1 g L^−1^), sodium acetate (5 g L^−1^) with a 1 mL drop of Tween 80, wherein it was maintained at pH 5.7, and autoclaved at 121 °C for 15 min. We stored the inoculated MRS agar Petri dishes at 37 °C for 16 h incubation. This composition was also established in our previous study [36]. Three consecutive MRS plates (streaked on four regions) were constructed to obtain pure bacterial colonies. The key properties observed in selecting the pure colonies were the color, shape, density, and margins after the re-streaking purification method. A month’s stock of collected isolates were sub-cultured and kept at −80 °C freezer in 20% glycerol (*v/v*) solution.

### 2.3. Hemolysis Test of Isolates

This study used the blood agar (hemolysis) test technique [37] to select bacterial isolates safe for fermenting the vanilla pods. Moreover, the strains can be used in animal and other agricultural studies or for handling during experiments. An infusion of 40 g of commercial agar-based media (Himedia Laboratories Co., Mumbai, Maharashtra, India) was added to distilled water (1 L) and then boiled to dissolve and autoclaved at 121 °C for 15 min at 15 lbs pressure for sterilization. A 5% (*v/v*) sterile sheep-defibrinated blood was added to cooled agar medium (45–50 °C) Petri dish plates. The pH was maintained at 7.3 ± 0.2 under room temperature lamina flow conditions. The isolated colonies were selected for the hemolysis test procedure on the blood agar culture and incubating the plates at 37 °C for 48 h. A lightbox (Hakuba, Japan) was used to view the hemolysis on the blood agar plates. The isolates showing gamma (γ) or no hemolysis were chosen since the bacteria did not ingest the blood agar. Agar plates with alpha (α) and (β) hemolysis were safely discarded and categorized as unsafe for food processing or human consumption [38].

### 2.4. Bacteria Identification and PCR Amplification

The selected bacteria isolates were then referred to Mission Biotech Taipei, Taiwan, for microbial identification. The researchers used a Qiagen DNeasy Plant Kit (Germantown, MD, USA) for DNA extraction. The purification and isolation identified the bacteria based on the 16S rRNA gene. The primer products were mixed according to the manufacturer’s instructions and quantified with the ABI 3730XL DNA Analyzer using the ABI Big Dye Terminator v.3.1 sequence reagent (Mission Biotech, Taipei, Taiwan). A 30.0 µL PCR mixture contained a PCR buffer of 30 µL, and 10 mM dNTP in 0.3 µL of template DNA. Preliminary denaturation was performed at 94 °C for 5 min. The denaturing and annealing steps were 40 cycles of 30 s at 94 and 55 °C, respectively. Moreover, the extension and final extension were performed separately at 2 min 20 s, and 5 min, respectively. According to this study, the primer set used for amplification on the 16S rRNA gene were F8 (5′-AGAGTTTGATCMTGGCTCAG-3′) and R1510 (5′-CGGTTACCTTGTTACGACTT-3′) [39] primers, respectively. The blast sequence of the 16S rRNA gene was amplified and submitted to the database obtained from the National Center for Biotechnology Information (NCBI) GeneBank.

### 2.5. Phylogenic Analysis of the Bacillus Strains Isolated from Vanilla

The neighbor-joining (NJ) method [40] was employed to construct the evolutionary relationships of taxa to better understand the neighboring *Bacillus* species per the pairs of operational taxonomic units. The evolutionary lengths were designed using the maximum composite likelihood technique [38] and presented numerically as base substitutions per site. Eleven (11) *Bacillus* nucleotides were used for the evolutionary analyses with MEGA11 software (version 11.0.13) [41], wherein the accession numbers can be obtained from the NCBI website: https://blast.ncbi.nlm.nih.gov/Blast (accessed on 13 June 2024) with a BLAST search. The phylogenetic tree was constructed based on the *Bacillus* isolates from *Vanilla planifolia*, confirmed with the NCBI database. Notably, three randomly selected *Bacillus* strains (used to ferment the vanilla samples) were marked for comparison during the construction of the phylogenetic tree.

### 2.6. Fermentation and Curing of Vanilla Beans

Newly harvested green vanilla bean pods from Pingtung farm were quickly blanched at a high temperature (80 °C) for 1 min as a killing procedure. The vanilla pods were then placed in dry tissue papers for 15 min to allow the pods to dry out. The prepared liquid culture of *Bacillus vallismortis* NR_104873.1:11-1518, *B. velezensis* ZN-S10, and *B. tropicus* KhEp-2 were subjected to UV/Vis spectrophotometer (DU 640, MicroDigital Co., Geumcheon-Gu, Seoul, South Korea) for absorbance measurement at OD _600_ = 1 (2 × 10^9^ CFU mL^−1^ = 1.0) following Rincón-Molina et al.’s [42] method. Each plastic box that had 1 kg of vanilla pods was sprayed with 1 mL of 1 × 10^7^ CFU mL^−1^ of the selected bacteria applied after the killing stage. The vanilla pods were then covered and shaken for 10–15 s to allow proper coating of the *Bacillus* sp. for effective sweating or fermentation of *V. planifolia*. The control samples were vanilla pods (1 kg) that were sweated or fermented conventionally i.e., without bacteria-coating. Studies have shown that sweating or fermentation is critical in activating *Bacillus* sp. in vanilla beans [22]. The conditioning and drying treatments were the same as the previously cured vanilla beans in this study. Hence, in this study after the bacteria-coating of the vanilla samples, they were stored for 3 weeks in a cold storage facility of 10 °C (Frigidaire Freezers Co., Kaohsiung, Taiwan) with an installed dehumidifier. The vanilla bean pods were also dried in this storage for 4 weeks. The conditioning stage involved storing the bacteria-treated pods in the same facility in plastic boxes until dark-brown, and cured vanilla pods (20% moisture content) were taken for further experiments. It should be noted that during the cold fermentation, drying, and conditioning of the vanilla involved evenly turning the beans every 48 h to avoid mold formation.

### 2.7. Determination of Volatile Compounds

Vanillin and volatile compounds extracted from cured vanilla beans (*V. planifolia*) were determined with methanol extraction for GC-MS analysis. The relative area percentage (%) of the volatile peaks was calculated from the peak area of the volatile per the total area of the peaks.

#### 2.7.1. Methanol Extraction

The vanilla pods were subjected to methanol extraction for the volatile compounds, wherein 1 g of vanilla was cut into 0.5 cm sizes. The samples were added to a 15 mL Falcon tube with 10 mL of 100% methanol and soaked overnight (18 h). The mixture was centrifuged at 12,000 rpm for 10 min at 4 °C. A supernatant of 1000 µL was then collected for GC-MS analysis.

#### 2.7.2. GC-MS Analysis

The GC-MS evaluations of the volatile components in different bacteria-treated vanilla were performed with a GC-MSD 5977 instrument series of GC-Agilent 7890B coupled with a GC-MSD Agilent 5977A (Shimadzu, Kyoto, Japan). An Agilent J & W DW-5MS UI of 30 m × 0.25 mm × 0.25 µm capillary column was used for the analysis. The heating column oven conditions were as follows: 40 °C, maintained for 0.5 min, then heated to 250 °C at a rate of 7 °C min^−1^ and kept constant for 10 min. A splitless injection method was used at 200 °C of inlet temperature. The sample was then transferred to an MSD column with an injection temperature of 280 °C, coupled with an Agilent G4513A automatic 10 µL liquid sampler, with a 1 µL syringe injection volume and no split. The mass spectrometry (MS) used an EI inert 35 ion source with a tandem axis detector where the solvent (toluene) and samples were injected at 3 min delays. Helium was employed as the carrier gas at a constant flow mode, 1.0 mL min^−1^ flow rate. The MS conditions were as follows: 250 °C ion source temperature, and MS Quadrupole temperature of 150 °C at a scanning range of 40 (*m/z*) to 450 (*m/z*) measured at TIC full scan mode. The spectra were identified and compared with the NIST11.L database. The qualitative analysis with more than 80% detection quality was presented for the final dataset of volatile compounds in different vanilla treatments.

## 3. Results and Discussion

### 3.1. Strain Isolation and 16S rRNA Identification

The bacteria culture was carried out on an MRS medium, resulting in 11 identified bacteria strains isolated from vanilla beans (*Vanilla planifolia*). Treating vanilla curing first involves blanching at high temperatures (65–70 °C). This procedure eliminates microorganisms, except thermophilic and thermotolerant *Bacillus* sp. bacteria, which accumulate during the growth of vanilla beans in the field [18]. Moreover, during the conventional curing process, *Bacillus* sp. has been reported to be responsible for the formation of vanillin due to glucovanillin hydrolysis [34] and thus was successfully isolated from traditionally cured *V. planifolia* pods. The isolates identified with 16S rRNA gene sequencing included *Bacillus tequilensis* AJM7, *B. vallismortis* NR_104873.1:11-1518, *B. tropicus* KhEp-2, *B. velezensis* ZN-S10, *Priesta megaterium* SF4, *B. velezensis* Ba-0321, *B. megaterium* HBUM06947, *B. licheniformis* GN02, *Acinetobacter pittii* SF6, *Bacillus sp.* cp64 and *B. subtilis* HSY21. According to the NCBI GeneBank database, the isolated bacteria were amplified on the 16S rRNA gene and showed a 100.0% identity of the *Bacillus velezensis* ZN-S10 (3,929,792 bp), *Bacillus tropicus* KhEp-2 and *Bacillus vallismortis* NR_104873.1:11-1518 strains. The strains KhEp-2 and NR_104873.1:11-1518 had partial sequences of 1510 bp and 1508 bp accession lengths, while ZN-S10 had 3,929,792 bp since it was the single candidate with a complete genome (Table 1). Therefore, based on these properties, the three *Bacillus* sp. strains were selected in this study to investigate their roles in vanilla fermentation during low temperatures of 10 °C.

### 3.2. Morphological Characteristics of the Bacillus Strains Isolated from Vanilla

**Figure 2:**
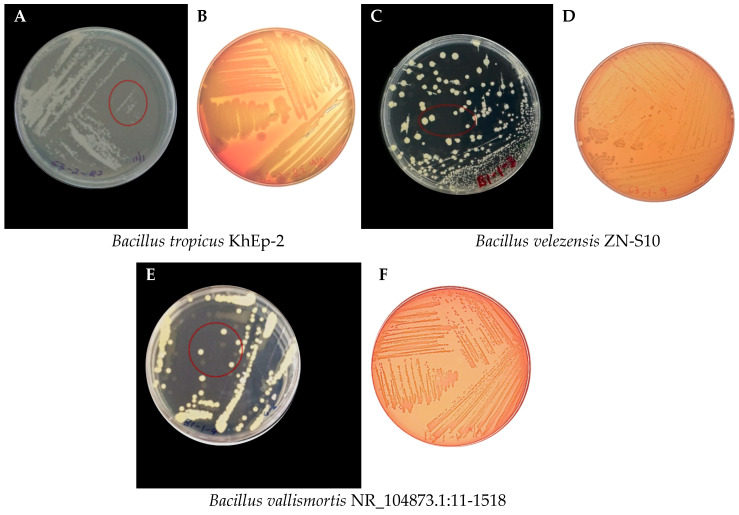
Morphological characteristics of isolated *Bacillus* strains from *Vanilla planifolia* that were cultured and grown on modified MRS agar medium plates and hemolysis agar plates of the *Bacillus* sp. strains isolated from *Vanilla planifolia* cultured at 37 °C on blood agar media for 48 h. The strains presented include *Bacillus tropicus* KhEp-2 (**A**,**B**), *Bacillus velezensis* ZN-S10 (**C**,**D**), and *Bacillus vallismortis* NR_104873.1:11-1518 (**E**,**F**) colonies on MRS and blood agar medium, respectively.

This study found that the isolates’ morphological characteristics were predominately *Bacillus* strains. The colonies were creamy-white in slightly oval to circular forms with regular margins (Figure 2) when grown after 16 h culture in MRS medium. For instance, the selected colonies to ferment vanilla beans showed that the colonies of *Bacillus tropicus* KhEp-2 (Figure 2A) had circular cells while *B. velezensis* ZN-S10 (Figure 2C) and *Bacillus vallismortis* NR_104873.1:11-1518 isolates (Figure 2E) had visibly round, cream-white, and fairly distributed colonies. These pure colonies were selected at the fourth region on the streaked plate (red circled in Figure 2A,C,E). The phenotypic properties observed in this study included endospore-forming rods, which also concurred with the *Bacillus vanillea* sp. nov. strain XY18^T^ that was isolated by Chen, Gu, Li, Xu, He and Fang [20] from cured vanilla beans. Hence, the modified MRS medium, also categorized as a *Lactobacilli* (LB) medium, can successfully culture and isolate *Bacillus* strains from vanilla beans. It should also be noted that the MRS medium was maintained at pH 5.7 for 16 h incubation at 37 °C, which may not be the optimal growth for these *Bacillus* isolates.

### 3.3. Hemolysis Physiognomies of the Bacillus Strains

The countenances of the hemolysis agar plates showed that the *Bacillus* strains (Figure 2) isolated from traditionally cured vanilla beans did not belong to *Streptococcus*, *Enterococcus*, and *Staphylococcus*, and hence were characterized to be safe for use in animal or human studies. The blood agar plates for *Bacillus tropicus* KhEp-2 (Figure 2B), *Bacillus velezensis* ZN-S10, and *Bacillus vallismortis* NR_104873.1:11-1518 (Figure 2D,F) showed that the bacteria grew well on the blood agar. The effective growth on the agar proved that there was no lysis of the red blood cells and gamma (γ) hemolysis was obtained. Therefore, these isolates were considered edible or safe for fermenting non-cured vanilla bean pods.

### 3.4. Phylogenic Tree of the Isolated Bacillus Strains from Vanilla

**Figure 3:**
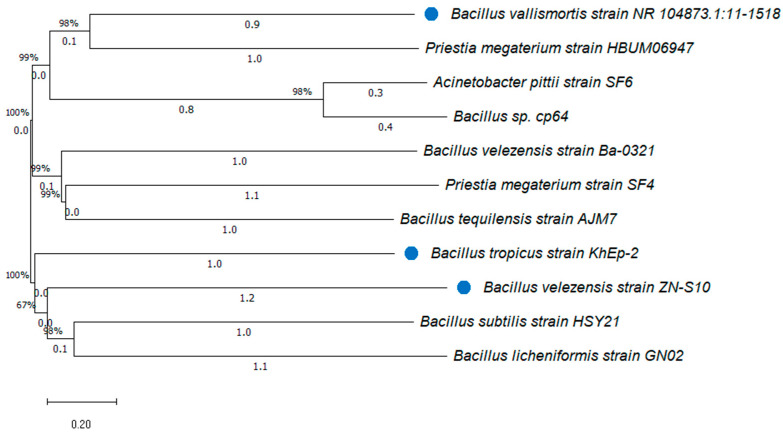
The neighbor-joining phylogenetic tree of the relationship among 11 *Bacillus* sp. strains isolated from vanilla beans (*V. planifolia*). The tree analyzed 11 nucleotide sequences evolutionarily using MEGA11 software (version 11.0.13). A pairwise deletion option on 1514 total positions was employed in the final dataset. Bar distance scale = 0.10.

According to the phylogenetic tree, the 16S rRNA gene sequencing comparison had 98% similarity between the *Bacillus* strains (Figure 3). *Bacillus* species have been reported as the dominant genus member throughout the curing process, as it has also been found in other studies [22]. Hence, from the 11 identified strains, we found that *Bacillus tropicus* strain KhEp-2 (99%) was the dominant species, closely related to *B. vallismortis* NR_104873.1:11-1518, *Priesta megaterium* HUM06947 strain with 98% similarity as well as to *Bacillus sp.* cp64 and *Acinetobacter pittii* strain SF6. Likewise, the phylogenic of the *B. subtilis* HSY21 strain was closely related to the *Bacillus* genus member in these isolates, with 98% sequence similarity to *B. licheniformis* GN02. The NJ method also exhibited that *B. velezensis* ZN-S10 bacteria were the predominant strains followed by the GN02 strain and *Priestia megaterium* SF4, with close sequence similarity to the HUM06947 strain, *B. velezensis* Ba-0321, *B. tequelensis* strain AJM7, *B. tropicus* KhEp-2 strain and *B. subtilis* HSY21. The *Bacillus* strains (as marked in Figure 3) that were randomly selected for coating to ferment the vanilla during the curing process included *B. vallismortis* NR_104873.1:11-1518, *B. tropicus* KhEp-2 strain, and *B. velezensis* ZN-S10. These strains were anticipated as bacterial candidates to improve the aromatic-volatile components of the vanilla pods used in this study.

### 3.5. Volatile Compounds Analysis by GC-MS

In this research, the volatile compounds were analyzed with gas chromatography-mass spectrometry (GC-MS) to evaluate the aroma and flavor profile alteration after spraying or coating vanilla pods with isolated bacteria strains for fermentation at 10 °C during the curing of *V. planifolia*. The dried-cured vanilla samples were collected from the cold storage and analyzed based on their methanol extracts. Zhang et al. [43] reported that low temperatures during *Baiju* fermentation reduced the undesired microbial population while increasing the volatile-flavor profile of fermented produce. Henceforth, the edible *Bacillus* sp. found in this research with 11 volatile compounds identified from bacteria-coated or fermented vanilla pods and 3 components were present in non-treated samples (Table 2).

#### 3.5.1. Gas Chromatography Profiles

**Figure 4:**
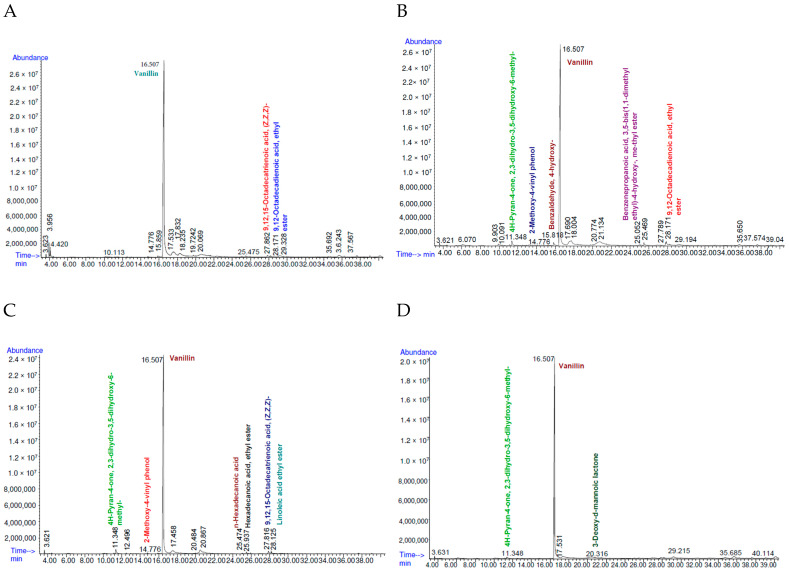
Gas chromatography of (**A**) non-bacteria-treated vanilla (control); (**B**) *Bacillus tropicus* KhEp-2-treated vanilla (**C**); *Bacillus velezensis* ZN-S10 treatment and (**D**) *Bacillus vallismortis* NR_104873.1:11-1518-coated vanilla beans, analyzed as methanol extract.

The total ion current (TIC) chromatography presented the abundance (x-axis) vs. the retention time in 1 min on the y-axis of the total mass range. The methanol extracts of the vanilla pods exhibited the three different bacteria-treated samples compared with the control group (non-bacteria-coated vanilla for fermentation). According to Figure 4, the methanol extract of non-treated vanilla (Figure 4A) had 19 peaks, similar to those detected with *Bacillus tropicus* KhEp-2 treated vanilla (Figure 4B). The GC-MS spectrum of *Bacillus velezensis* ZN-S10-fermented vanilla treatment (Figure 4C) had 11 peaks while *B. vallismortis* NR_104873.1:11-1518-coated vanilla beans had 7 peaks (Figure 4D). The aromatic components were 11 for all the bacteria treatments, compared to 3 compounds in the control samples. In another study, the GC-MS analysis of methanol extract reported nine constituents from Indian vanilla beans [25]. Vanillin peak was the highest peak at the retention time (RT) of 16.507 min for the control group, *Bacillus tropicus-* and *B. velezensis*-coated samples, as well as for the *B. vallismortis*-treated samples. The relative area (RA) percentages were quantitively analyzed per the total area peaks (Supplementary data). In this study, the GC-MS technique identified three compounds in the methanol extract of *B. vallismortis*-treated vanilla. These components comprised 4H-pyran-4-one,2,3-dihydro-3,5-dihydroxy-6-methyl (1.16% RA), vanillin (87.4% RA), and 3-Deoxy-d-mannoic lactone (0.23% RA) (Table S1). The lactone was denoted as a new cyclic ester found in vanilla, and studies exhibited it as a key constituent for flavor contribution in fermented fruits [44]. Remarkably, and according to the published vanilla studies, 3-Deoxy-d-mannoic lactone has not been reported in vanilla. The presence of lactone showed effective fermentation with the *B. vallismortis* NR_104873.1:11-1518 strain, which might also improve the flavor attributes of *V. planifolia*. Linoleic acid ethyl ester at the RT of 28.163 min was only found in the *B. velezensis* ZN-S10 strain treatment. Moreover, a 0.34% area (Table S2) was yielded on the RT of Linoleic acid ethyl ester which was shown to influence the volatile profile of *V. planifolia* relatively fairly, rather than vanillin contribution noted with 85.8 RA% of vanillin on the ZN-S10 treatment. These findings also confirmed that edible *Bacillus* strains can be used to alter the aromatic properties of vanilla. However, this study’s interest requires future research. It is also worth noting that the 4H-pyran-4-one, 2,3-dihydro-3,5-dihydroxy-6-methyl compound was detected only in bacteria-treated vanilla pods, with RT*s* of 11.348 min. Studies have shown that this component belongs to the ketone groups formed due to the Maillard reaction that will result in browning and increased aromatic characteristics [45,46]. Hereafter, the vanilla samples were effectively fermented with produced, brown-cured pods. Discernibly, 9,12-Octadecadienoic acid, ethyl ester, and 9,12,15-Octadecatrienoic acid (Z,Z,Z) were the second highest peaks found in the vanilla samples suggesting better aroma quality of *V. planifolia*. The retention time of 9,12,15-Octadecatrienoic acid (Z,Z,Z) was 27.862 min for *B. velezensis,* acquiring 0.97% area (Table S2) after vanillin as the main compound found in *V. planifolia*. However, these components were not found in *B. vallismortis* treatment. The RT of 9,12-Octadecadienoic acid, ethyl ester, was 27.862 min found in non-coated bacteria and *B. tropicus* KhEp-2-fermented vanilla. Relatively, vanillin varied between the two treatments of the vanilla bean pods. A 76.8% area of vanillin was obtained in KhEp-2-coated vanilla (Table S3), while 89.0% RA was found in non-treated samples (Table S4). The GC-MS analysis also showed that seven compounds were found in the ZN-S10 treatment, while six compounds were present in KhEp-2-coated vanilla followed by *B. vallismortis* and the control group (three components). The results showed that bacteria treatment improved the volatile profile of *V. planifolia*, as found in *B. tropicus* KhEp-2 and *B. vallismortis* NR_104873.1:11-1518 fermentation at 10 °C.

#### 3.5.2. Analysis of Volatile Compounds

GC-MS analysis of the volatile components of the *V. planifolia* pods from non-bacterial-treated samples (control), *Bacillus tropicus* KhEp-2-treated vanilla, *B. velezensis* ZN-S10, and *B. vallismortis* NR_104873.1:11-1518 treatment through methanol extraction showed 11 volatile compounds, as presented in Table 2. The NIST Chemistry WebBook, SRD 69 database https://www.nist.gov/ (accessed on 29 April 2024), from NIST11.L was applied for the compounds’ identification, naming, and comparison of the GC-MS spectra. Notably, volatile compounds with less than 80% GC-MS quality detection were not shown (presented in Supplementary Material). Vanillin content as the key aroma component was detected in all vanilla pods used in this research. The vanilla pods that were allowed to ferment conventionally (control) had a qualitatively 96% score of vanillin, which was also detected in *B. vallismortis*-coated vanilla and *B. velezensis* ZN-S10-treated pods. Similarly, high vanillin content of qualitatively 83% was detected with GC-MS from Taiwanese, Taoyuan Longtan vanilla samples treated with edible *B. subtilis*. Chen, Gu, Li, Xu, He and Fang [20] reported that treating vanilla with *Bacillus* strains (*B. subtilis* XY20 and *B. vanillea* XY18) resulted in high vanillin compared to non-coated vanilla pods. However, Chen, Lin, Lo and Hsu [33] exhibited that *B. subtilis subsp. subtilis* treated on vanilla yielded 30.22% of vanillin’s relative area percentage recorded at early curing stages. In contrast, in this study, the analysis was when the vanilla pods were at the late drying curing process (15% dry weight). This study found that more volatile components were identified in *B. velezensis* ZN-S10-treated vanilla followed by the samples coated with the *Bacillus tropicus* KhEp-2 strain. As compared with the control group, the ketone compound—4H-pyran-4-one, 2,3-dihydro-3,5-dihydroxy-6-methyl—was detected in all bacteria-treated vanilla pods at 11.348 min of retention time, wherein a qualitative score of 90% in *B. velezensis* ZN-S10-treated vanilla pods, as well as in *B. tropicus*, and *B. vallismortis* bacteria-coated groups. However, this compound was not found in non-bacteria-fermented vanilla. Studies have shown that the 4H-pyran-4-one, 2,3-dihydro-3,5-dihydroxy-6-methyl- metabolite has been predominantly in fermented products with *Saccharomyces cerevisiae* [47] showing effective fermentation of the vanilla pods with the isolated pure bacteria strains. In this research, 9,12-octadecadienoic acid, ethyl ester (97%) was only found in non-bacteria-coated vanilla. These findings concurred with the methanol extract from Indian cured vanilla pods, that were not fermented with *Bacillus* isolates, where in 9,12-octadecadienoic acid, ethyl ester was also detected [25]. Moreover, the compound was relatively detected in *B. tropicus*-treated vanilla samples. It should also be noted that benzaldehyde, 4-hydroxy- and benzenepropanoic acid, 3,5-bis(1,1-dimethyl ethyl)-4-hydroxy-, methyl ester were detected only with *B. tropicus* KhEp-2-treated vanilla. Hence, the vanilla volatile characteristics had more components in *B. tropicus* KhEp-2 treated vanilla compared to non-bacteria samples that had vanillin, 9,12,15-Octadecatrienoic acid, (Z,Z,Z) and 9,12-Octadecadienoic acid, ethyl ester. Benzaldehyde, 4-hydroxyl was qualitatively 93% in KhEp-2 strain treatment on *V. planifolia*, which has been exhibited as a common qualitative score range (93–96%) by other studies for this vanilla cultivar [17]. Notably, *B. tropicus* KhEp-2- and *B. velezensis* ZN-S10-treated vanilla had 2-Methoxy-4-vinylphenol, which has been attributed to playing a role as a vanillin precursor as reported by van Schijndel et al. [48] in another study. Hence, the results suggested that isolated pure *Bacillus* strains could be used to alter a better vanillin profile in vanilla. The methanol extracts of *V. planifolia* also exhibited an 89% quality score of 3-Deoxy-d-mannoic lactone with *B. vallismortis* treatment which can serve the vanilla with phalsa cherry (*Grewia tenax*) odor [49]. McCormick [50] detected the 3-deoxy-d-mannoic lactone component with 25% ethanol extract from vanilla beans, which was similar to the findings of this study. The GC-MS analysis showed *B. vallismortis* NR_104873.1:11-1518-treated vanilla pods could identify three major volatile components (4H-Pyran-4-one, 2,3-dihydro-3,5-dihydroxy-6-methyl-, Vanillin, and 3-Deoxy-d-mannoic lactone), which were fewer in comparison to the other two strains’ treatment. Hence, this *Bacillus* strain might not increase the vanilla flavor and aroma properties. These findings also show the further need for research on the bacteria-curing technique on vanilla beans with an application of other strains. The fatty acids, 9,12,15-Octadecatrienoic acid, (Z,Z,Z) and n-Hexadecanoic acid, were detected in *B. velezensis* ZN-S10-treated vanilla at 99% and 98% qualitative scores, respectively. Other researchers have reported that these compounds were present in *V. planifolia* Andrew [51] and other Orchidaceae plant species [52]. The study also found that Linoleic acid ethyl ester and Hexadecanoic acid, ethyl ester detected only *B. velezensis*-fermented vanilla pods. There were seven volatile compounds identified with the *B. velezensis* ZN-S10 and *B. tropicus* KhEp-2 strains showing an increased volatile profile, thus the bacterial fermentation improved the vanilla quality.

## 4. Conclusions

In this research, 11 *Bacillus* strains were effectively isolated from *Vanilla planifolia* pods identified through morphological observation and 16S rRNA gene sequencing. The *Bacillus* sp. isolates were considered thermophilic and thermoresistant due to their survival during the blanching process performed in this study. The bacteria treatment or coating with *B. tropicus* KhEp-2, *B. velezensis* ZN-S10, and *B. vallismortis* NR_104873.1:11-1518 strains isolated from conventionally fermented vanilla pods effectively fermented green-blanched vanilla (which were later fermented in a cold storage at 10 °C for two weeks). The cold fermentation also eliminated spoilage microorganisms, resulting in cured vanilla pods with an increased aroma profile that suggests a better vanilla quality when observed with the volatile profile detected by the GC-MS technique. The novel 3-Deoxy-d-mannoic lactone compound was detected in *B. vallismortis*-treated samples, showing that an alteration of aroma or volatile compounds on vanilla beans could be performed through edible microorganisms. The two strains, ZN-S10 and KhEp-2, were considered the best candidates owing to the more volatile compounds found in the methanol extracts of *V. planifolia* compared to the *B. vallismortis*-treated vanilla and the control. Moreover, the bacteria-fermented vanilla pods had higher vanillin than non-treated vanilla. An effective curing process of vanilla with bacteria fermentation during the sweating stage at low temperature sustainably saved energy use, fulfilling sustainable development goal 7. The low temperature of 10 °C used in this study for fermentation was due to the winter climatic conditions (cool and wet humidity) in vanilla bean pods in Taiwan. These conditions are suitable for the fermentation of *V. planifolia* since less heat loss is required during the curing process; hence saving energy consumption. This is contrary to vanilla bean pods from Madagascar [1] where the conditions are warm and dry, which requires more energy to ferment the bean pods. Henceforth, this study also showed that applying edible bacteria for fermentation or sweating at low temperatures is essential for improved fermentation, curing, and quality control of vanilla beans for food flavoring processes.

## Figures and Tables

**Table 1:** The details of the isolated bacterial strains selected for vanilla fermentation.

Sample Code	Strain Name	Identity (%)	Accession Length (bp)	Accession ID	Genome Sequence
C3-2-R2	*Bacillus tropicus* KhEp-2	100.00	1510	OP422217.1	partial
C3-1-9	*Bacillus velezensis* ZN-S10	100.00	3,929,792	CP102933.1	complete
B1-1-5	*Bacillus vallismortis* NR_104873.1:11-1518	100.00	1508	OP104906.1	partial

**Table 2:** The GC-MS qualitative analysis of the major volatile components from the methanol extract of three different *Bacillus*-treated vanilla pods.

					Treatment			
Compounds	RT (min)	Qualitative Score (%)	Mol Weight (amu)	CAS No.	Control	*B. tropicus* KhEp-2	*B. velezensis* ZN-S10	*B. vallismortis* NR_104873.1:11-1518
(1) 4H-Pyran-4-one, 2,3-dihydro-3,5-dihydroxy-6-methyl-	11.348	90	144.042	028564-83-2	-	✓	✓	✓
(2) 2-Methoxy-4-vinyl phenol	14.776	91	150.068	007786-61-0	-	✓	✓	-
(3) Benzaldehyde, 4-hydroxy-	15.816	93	122.037	006386-38-5	-	✓	-	-
(4) Vanillin	16.507	96	152.047	000121-33-5	✓	✓	✓	✓
(5) 3-Deoxy-d-mannoic lactone	20.316	89	162.053	1000127-87-1	-	-	-	✓
(6) n-Hexadecanoic acid	25.474	98	256.240	000057-10-3	-	✓	✓	-
(7) Hexadecanoic acid, ethyl ester	25.937	96	278.272	000628-97-7	-	-	✓	-
(8) Benzenepropanoic acid, 3,5-bis(1,1-dimethyl ethyl)-4-hydroxy-, methyl ester	25.052	87	292.204	006386-38-5	-	✓	-	-
(9) 9,12,15-Octadecatrienoic acid, (Z,Z,Z)-	27.862	99	278.225	000463-40-1	✓	-	✓	-
(10) 9,12-Octadecadienoic acid, ethyl ester	28.171	97	280.24	007619-08-1	✓	✓	-	-
(11) Linoleic acid ethyl ester	28.163	99	308.272	000544-35-4	-	-	✓	-

✓, detected; -, not detected; RT, retention time; and CAS no., Chemical Abstracts Service number.

## Data Availability

The data presented in this article are available in the NPUST library database.

## Supplementary Materials

The following are available online at https://www.mdpi.com/article/10.3390/foods13172777/s1, Table S1. Specific GC-MS details of *Bacillus vallismortis* NR_104873.1:11-1518 treated vanilla samples, Table S2. Specific GC-MS details of *Bacillus velezensis* ZN-S10 treated vanilla samples, Table S3. Specific GC-MS details *Bacillus tropicus* KhEp-2 treated vanilla samples, Table S4. Specific GC-MS details of non-treated vanilla samples (the control group).
