# Supplementary material for: Exploring the Volatile Profile of Vanilla planifolia after Fermentation at Low Temperature with Bacillus Isolates
Source: Foods. 2024 Aug 30;13(17):2777. doi: 10.3390/foods13172777 (PMC11394893; doi:10.3390/foods13172777)
Supplement: Supplementary file 1 [file foods-13-02777-s001.zip › foods-3137403-supplementary.pdf]

Supplementary Data

Table S1. Specific GC-MS details of *Bacillus vallismortis* NR_104873.1:11-1518 treated vanilla samples

RT (min)	Area (Ab*s)	Relative Area (%)	Baseline Heigth (Ab)	Absolute Heigth (Ab)	Peak Width 50% (min)	Compound Name	Quality (%)	Mol Weight (amu)
11.348	12603395	1.16941	151054	226710	0.109	4H-Pyran-4-one, 2,3-dihydro-3,5-dihydroxy-6-methyl-	90	144.042
16.507	9.42E+08	87.37744	20372988	20472849	0.061	Vanillin	96	152.047
17.351	21879819	2.03012	166930	313587	0.162	4H-Pyrazole, 3-tert-butylsulfanyl-4,4-bistrifluoromethyl-	10	292.047
17.538	8852242	0.82135	99622	256320	0.113	Vanillin	83	152.047
20.316	2479628	0.23007	13285	227331	0.224	3-Deoxy-d-mannoic lactone	89	162.053
29.219	24932944	2.31340	141956	258106	0.223	Cyclopentanecarboxamide, N-(4-fluorophenyl)-	53	207.106
35.685	4013	3.35910	211542	317167	0.207	2H-Pyran, 2-(8-dodecynyloxy)tetrahydro-	38	266.225
40.112	4558	2.69911	156324	270022	0.234	Kauren-18-ol, acetate, (4.beta.)-	14	330.256
		100						

Note: Compounds with less than 80% Quality detection were not shown in the final data set (Table 2).

Table S2. Specific GC-MS details of *Bacillus velezensis* ZN-S10 treated vanilla samples

RT (min)	Area (Ab*s)	Relative Area (%)	Baseline Heigth (Ab)	Absolute Heigth (Ab)	Peak Width 50% (min)	Compound Name	Quality (%)	Mol Weight (amu)
11.348	37664058	2.196593	538196	637105	0.094	4H-Pyran-4-one, 2,3-dihydro-3,5-dihydroxy-6-methyl-	90	144.042
12.493	4020000	0.234449	104271	194193	0.053	Dodecane	60	170.203
14.776	4432697	0.258518	117645	216733	0.054	2-Methoxy-4-vinyl phenol	91	150.068
16.507	1.47E+09	85.83878	24151243	24270722	0.08	Vanillin	96	152.047
17.489	30806563	1.796659	312329	496343	0.124	Propanoic acid, 3-bromo-, ethyl ester	12	179.979
17.53	32823050	1.914262	307154	494106	0.178	Lethane	10	203.098
20.486	77075557	4.495098	411333	563993	0.247	.beta.-l-Arabinopyranoside, methyl	43	164.068
20.868	20561989	1.199189	157059	316792	0.161	Benzene ethanamine, 2,5-dimethoxy-.alpha.-methyl-	35	195.126
25.474	10626333	0.619735	196163	301661	0.073	n-Hexadecanoic acid	98	256.24
25.937	2206346	0.128676	112100	222534	0.031	Hexadecanoic acid, ethyl ester	96	284.272
27.862	16754422	0.977129	304367	429363	0.074	9,12,15-Octadecatrienoic acid, (Z,Z,Z)-	98	278.225
28.163	5845553	0.340917	297030	469914	0.031	Linoleic acid ethyl ester	99	308.272
	1.73E+09	100						

Note: Compounds with less than 80% Quality detection were not shown in the final data set (Table 2).

Table S3. Specific GC-MS details *Bacillus tropicus* KhEp-2 treated vanilla samples

RT (min)	Area (Ab*s)	Relative Area (%)	Baseline Heigth (Ab)	Absolute Heigth (Ab)	Peak Width 50% (min)	Compound Name	Quality (%)	Mol Weight (amu)
6.068	24189786	1.061608	145946	294862	0.203	3-Amino-2-oxazolidinone	43	102.043
9.902	15966888	0.700732	235562	368925	0.094	1,3,5-Triazine-2,4,6-triamine	72	126.065
10.097	6284439	0.275803	101711	260338	0.085	1H-Imidazole-4-carboxamide, 5-amino-	47	126.054
11.104	7601471	0.333603	128138	266732	0.087	Ethanamine, N-ethyl-N-nitroso-	59	102.079
11.348	39904398	1.751269	626956	784348	0.089	4H-Pyran-4-one, 2,3-dihydro-3,5-dihydroxy-6-methyl-	90	144.042
14.776	3677418	0.161389	99512	262205	0.053	2-Methoxy-4-vinylphenol	91	150.068
15.816	27723058	1.216671	419986	576197	0.09	Benzaldehyde, 4-hydroxy-	93	122.037
16.507	1749270795	76.769555	26651986	26846733	0.084	Vanillin	96	152.047
17.692	128315079	5.631313	508978	794048	0.306	Chloroacetic acid, 2,2-dimethylpropyl ester	16	164.06
18.001	2691755	0.118132	62480	371881	0.072	Guanosine	47	283.092
20.771	124636384	5.469868	355701	671204	0.422	2R,3S-9-[1,3,4-Trihydroxy-2-butoxymethyl]guanine	38	285.107
21.136	7056111	0.309669	110155	465885	0.107	3-Deoxy-d-mannoic lactone	64	162.053
25.052	2723192	0.119512	103290	293763	0.039	Benzenepropanoic acid, 3,5-bis(1,1-dimethylethyl)-4-hydroxy-, methyl ester	87	292.204
25.466	19154959	0.840646	375075	560782	0.069	n-Hexadecanoic acid	99	256.24
27.789	11841420	0.519680	404700	581672	0.043	9,12-Octadecadienoic acid (Z,Z)-	99	280.24
27.862	33147259	1.454721	526429	711903	0.083	9,12-Octadecadienoic acid (Z,Z)-	97	280.24
28.171	7214896	0.316637	131186	353418	0.073	9,12-Octadecadienoic acid (Z,Z)-	97	280.24
29.194	21131633	0.927396	119527	321361	0.215	Cyclopropyl 2-thienyl ketone	43	152.03
35.652	25223471	1.106972	145895	299111	0.217	Nonadecane-2,4-dione	38	296.272
37.577	13278029	0.582728	172048	306532	0.107	Cyclopentanecarboxamide, N-(2-fluorophenyl)-	53	207.106
39.048	7567184	0.332098	99931	233074	0.107	Tetrahydropyran-2-yl Z,Z-8,10-dodecadienoate	42	280.204
	2287838184	100						

Note: Compounds with less than 80% Quality detection were not shown in the final data set (Table 2). An average of qualitative percentage of 9,12-Octadecadienoic acid (Z,Z)- was used for the final presented data.

Table S4. Specific GC-MS details of non-treated vanilla samples (the control group)

RT (min)	Area (Ab*s)	Relative Area (%)	Baseline Heigth (Ab)	Absolute Heigth (Ab)	Peak Width 50% (min)	Compound Name	Quality (%)	Mol Weight (amu)
11.348	17464096	1.83257	188141	256063	0.124	4H-Pyran-4-one, 2,3-dihydro-3,5-dihydroxy-6-methyl-	58	144.042
16.466	8.48E+08	89.02938	19336845	19428535	0.06	Vanillin	96	152.047
17.44	73794351	7.743505	332968	448644	0.268	5-Tridecanone	10	198.198
27.862	12016475	1.260932	195508	278013	0.083	9,12,15-Octadecatrienoic acid, (Z,Z,Z)-	99	278.225
28.171	1273330	0.133615	62456	185404	0.032	9,12-Octadecadienoic acid, ethyl ester	97	308.272
	9.53E+08	100						

Note: Compounds with less than 80% Quality detection were not shown in the final data set (Table 2).
